# Association between expression of ZBP1, AIM2, and MDA5 genes and severity of COVID-19

**DOI:** 10.17179/excli2022-5141

**Published:** 2022-09-01

**Authors:** Nasir Arefinia, Amin Ramezani, Mehrdad Farokhnia, Ali Mohammad Arab Zadeh, Ramin Yaghobi, Jamal Sarvari

**Affiliations:** 1 Department of Bacteriology and Virology, School of Medicine, Shiraz University of Medical Sciences, Shiraz, Iran; 2 Shiraz Institute for Cancer Research, School of Medicine, Shiraz University of Medical Sciences, Shiraz, Iran; 3 Department of Medical Biotechnology, School of Advanced Medical Sciences and Technologies, Shiraz University of Medical Sciences, Shiraz, Iran; 4 Department of Internal Medicine, School of Medicine, Research Center for Hydatid Disease in Iran, Afzalipour Hospital, Kerman University of Medical Sciences, Kerman, Iran; 5 Transplant Research Center, Department of Pathology, Shiraz University of Medical Sciences, Shiraz, Iran; 6 Gastroenterohepatology Research Center, Shiraz University of Medical Sciences, Shiraz, Iran

**Keywords:** ZBP1, AIM2, MDA5, SARS-CoV-2, COVID-19, severity of COVID-19

## Abstract

Antiviral and inflammatory responses following the detection of the virus genome by nucleic acid sensors play a vital role in the pathogenesis and outcome of diseases. In this study, we investigated the ZBP1, AIM2, and MDA5 expression levels in COVID-19 patients with different intensities of the disease. 75 quantitative Real-Time PCR (qRT-PCR)-confirmed COVID-19 patients were included consecutively and divided into 3 groups of mild, severe, and critical based on the severity of the disease. Also, 25 healthy volunteer subjects were included. PBMCs were collected from the whole blood, and RNA was extracted using commercial kit. The expression of ZBP1, AIM2, and MDA5 genes was investigated using qRT-PCR technique. The mean age of the patients and healthy volunteers was 52.73±13.78 and 49.120±12.490, respectively. In each group, 13 out of 25 participants were male. The expression levels of ZBP1 (P=0.001), AIM2 (P=0.001), and MDA5 (P= 0.003) transcript were significantly higher in COVID-19 patients than the control group. The results also revealed that the expression levels of ZBP1, AIM2, and MDA5 were significantly higher in the critical and severe COVID-19 patients compared to those with mild disease (P<0.05). Moreover, regarding the gender, the expression levels of AIM2 and MDA5 were significantly elevated in male severe (P=0.04 and P=0.003, respectively) and critical (P=0.005 and P=0.0004, respectively) patients than the female ones. The results indicated that ZBP1, AIM2, and MDA5 genes might have an important role in the severity of COVID-19 disease. Moreover, the severity of COVID-19 disease in male and female patients might be related to AIM2, and MDA5 expression levels. More studies are recommended to be conducted to clarify this issue.

## Introduction

The SARS-CoV-2 virus has caused a global pandemic in 2020, infecting millions of people and leading to a global health crisis. COVID-19, a disease caused by SARS-CoV-2, causes not only severe pneumonia and acute respiratory distress syndrome (ARDS) in vulnerable people, but also damage to many organs, including the heart, kidney, pancreas, etc. (Sharif-Zak et al., 2022[37]). It has devastating health effects with high mortality and morbidity worldwide (Guan et al., 2020[11]). The disease is still a mystery and many questions about its pathogenesis remain unanswered. As it is clear, the immune system has an important role in its pathogenicity. Host defense in mammals fights the pathogens through two types of immunity: innate and adaptive immunity (Chaplin, 2010[5]). Intrinsic immunity acts as a pathogenic sensor and helps to eradicate the pathogens and build compatible immunity. These functions are highly dependent on pattern recognition receptors (PRRs) (Ansariniya et al., 2021[2]). PRRs are expressed in many cell types throughout the body and are, therefore, the first sensors likely to detect many viral infections, including SARS-CoV-2 (Kang et al., 2002[18], 2004[17]). 

One of the most important interferon-induced PRRs is Z-DNA-binding protein 1 (ZBP1), which functions as a cytosolic nucleic acid sensor and an antiviral immune response modulator (Daniels et al., 2019[7]; Ghoreshi et al., 2022[10]). After activation, ZBP1 recruits the receptor interacting protein kinase (RIPK3) 1 and 3 to execute programmed cell death, including apoptosis, necroptosis, pyroptosis or a mixture of them, depending on the cell type and caspase activity (Thapa et al., 2016[39]). ZBP1 promotes inflammatory signaling and contributes to SARS-CoV-2-induced cytokine production (Bader et al., 2022[3]). It has been reported that ZBP1 proteins are able to induce cell death in response to influenza A virus (IAV) infection (Zhang et al., 2020[50]). 

One of cytoplasmic immune system sensors which plays a key role in inflammation is Absent In Melanoma 2 (AIM2) (Junqueira et al., 2021[15]). AIM2 has antiviral responses by sensing the genome DNA of a host mitochondria, the membrane of which was damaged during pyroptosis (Wang et al., 2020[42]). SARS-CoV-2-infected cells undergo programmed cell death mediated by AIM2 inflammasome (Junqueira et al., 2022[14]). In addition, it induces cytokine storm through production of remarkably high level of inflammatory cytokine and eventually reduces the immune response (Liu et al., 2021[23]).

Another cytoplasmic immune system sensor is MDA5. Although first identified in the context of cancer (Kang et al., 2004[17]), it has been shown to have several roles in the host defense against a wide variety of viruses such as coronavirus (Roth-Cross et al., 2008[35]). MDA5 could detect different conformations of RNA, and it is critical for inducing type I interferon (IFN-I) (Zalinger et al., 2015[47]). It has been reported that cytosolic MDA5 is crucial to the induction of IFN type I and III in lung cancer cells following SARS-CoV-2 infection (Sampaio et al., 2021[36]). Moreover, the mice deficient in MDA5 expression demonstrated greater severity of disease when infected with MHV than did wild-type mice (Zalinger et al., 2015[47]).

Therefore, in this study we aimed to compare the expression levels of ZBP1, AIM2, and MDA5 in different stages of COVID-19 disease as well as the healthy control group.

## Materials and Methods

### Subjects

From February to March 2021, 75 COVID-19 patients were recruited consecutively from Afzalipour Hospital affiliated to the Kerman University of Medical Science. The selected patients were divided into three groups including critical (n=25), severe (n=25), and mild (n=25) according to the World Health Organization (WHO) criteria (WHO, 2021[45]). Inclusion criteria were as follows: 1) patients that met the diagnostic standard of SARS CoV-2 virus based on Centers for Disease Control and Prevention (CDC) definition, as an acute respiratory disease with laboratory-confirmed SARS CoV-2 infection by qRT-PCR (WHO, 2021[45]); 2) Positive results from throat swabs; 3) Patients with intense cough with bloody sputum, purulent sputum, chest pain, severe vomiting, diarrhea, dehydration and dyspnea; 4) Patients with complete clinical information; 5) Patients who did not complain about other infectious diseases, history of hematological diseases or immune system disorders; and 6) Patients who were inoculated with any of the Corona vaccines. In addition, patients under the age of 26 or over 76, pregnant women, and patients with the underlying disorder were excluded from the study. Moreover, 25 age- and gender-matched volunteers, as a healthy control group, were also included. Exclusion criteria for the healthy control group included any underlying chronic viral diseases such as HIV, HBV and HCV, cancers, autoimmune diseases as well as recent bacterial and/or viral infections. A structured questionnaire was used to collect demographic characteristics, temperature, and number of breaths per minute, weekly exercise, smoking, and history of underlying disease. Written informed consent was obtained from each participant, and the study was approved by the local Ethics Committee of Shiraz University of Medical Sciences (IR.SUMS.REC.1400.737).

### Criteria for the severity of COVID-19

Clinically, patients with the following symptoms were considered critical: 1) continuous high fever for 3 days or more; 2) intense cough with bloody sputum, purulent sputum, or chest CT; 3) rapid respiratory rate, dyspnea and cyanosis in the lips; 4) mind changes including unresponsiveness, somnolence, restlessness, and convulsion; 5) intense vomiting, diarrhea and dehydration; 6) signs of pneumonia examined by radiography; and 7) rapidly increased levels of Lactate dehydrogenase (LDH) and D-Dimer. In our study, patients who met the diagnostic criteria for SARS CoV-2 and also met three of the above conditions were assigned to the severe COVID-19 group (25 cases), and those who met the diagnostic criteria for SARS CoV-2 but not the above conditions were included in the mild COVID-19 group (25 cases).

### Blood sampling and PBMC isolation

Totally, 6 mL of blood was collected from each participant in heparin tube. Peripheral Blood Mononuclear Cells (PBMCs) were immediately prepared from the six mL blood diluted with phosphate‐buffered saline (PBS), using Ficoll (Bahar-Afshan, Iran) by the standard density gradient centrifugation, followed by two washes with PBS. Isolated PBMCs pellets were re-suspended in 200 μL of PBS. The cells were counted by a hemocytometer (Neubauer chamber). Aliquots of approximately 2.1 million cells were stored at -80 °C until further analysis.

### RNA extraction and real time-PCR

RNA was extracted from PBMCs using the commercial Kit (Favorgen, Taiwan), according to the manufacture instructions. The absorbance of the extracted RNAwas measured at 280 and 260 nm wavelength, using a Nanodrop ND 1000 (Thermo Scientific USA) to determine their quantity and purity. The extracted RNA samples were converted to complementary DNA (cDNA), using cDNA synthesis kits (Yekta-Tajhiz, Iran) according to the manufacturer's instructions. qRT-PCR system (QIAGEN Systems, Germany) was utilized to assess the ZBP1, AIM2, and MDA5 gene expression using SYBR green master mix (Amplicon, United Kingdom). qRT-PCR amplifications with 96 to 106 % efficiencies were set up in triplicate with a 10 μL volume containing 5 μL SYBR green master mix, 1 μL of the corresponding cDNA, and 10 pMol primers (Table 1(Tab. 1)). Amplification efficiencies were calculated and included in data normalization. The qRT-PCR was performed at the following conditions: 94 °C for 15 min; 40 sequential cycles at 95 °C for 10 s, 59 °C, 58.5 °C, 60.4 °C (ZBP1, AIM2 and MDA5, respectively) for 30 sec; and at 72 °C for 25 sec. At 72 °C for five min, the final extension was completed. Analysis of the melt curve confirmed the specificity of qRT-PCR. The β-actin gene was also used as an internal control and the levels of the ZBP1, AIM2, and MDA5 expression were calculated by Pfaffl formula. 

### Statistical analysis

The SPSS 26 and graphPad Prism 9 software packages were used for data analysis and drawing the graphs. Data were analyzed using independent-samples t-test, nonparametric test following Dunnett's test and two-way ANOVA. P values less than 0.05 were considered as significant.

## Results

### Demographic characteristics of the subjects

In this cross-sectional study, 100 subjects which included 75 COVID-19 patients (25 mild, 25 severe and 25 critical) as well as 25 healthy volunteer subjects participated in this study (Table 2(Tab. 2)). Of the 25 subjects in each group, 13 were male and 12 were female. The mean ages of the mild, severe, and critical patients and healthy volunteer group were 52.84±2.70, 51.08±2.40, 54.28±2.44, and 49.12±2.75, respectively (Table 2(Tab. 2)).

### Expression of ZBP1, AIM2 and MDA5 genes in COVID-19 patients and healthy subjects

**Figure 1:**
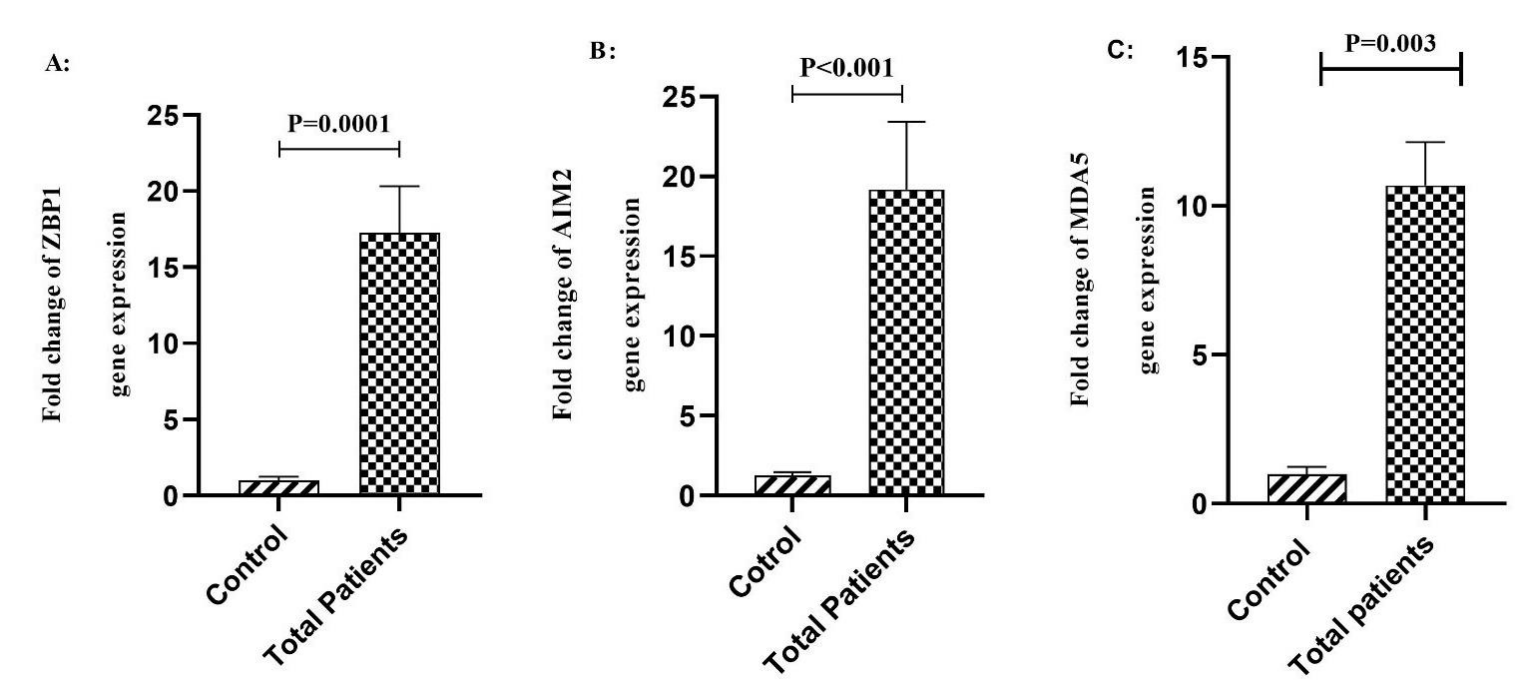
A) Comparison of the expression of ZBP1 in the peripheral blood samples between total COVID-19 patients and the control group. B) Comparison of the expression of AIM2 in the peripheral blood samples between total COVID-19 patients and the control group. C) Comparison of the expression of MDA5 in the peripheral blood samples between total COVID-19 patients and the control group. Data are presented as fold change. ns: not significant. Significant difference in different groups (p < 0.05).

Our results showed a significant increase in the expression level of ZBP1 (P=0.0001), AIM2 (P<0.001), and MDA5 (P=0.003) genes in COVID-19 patients compared to the healthy control group (Figure 1(Fig. 1)).

### Expression of ZBP1, AIM2 and MDA5 genes in COVID-19 patients based on the outcome of the disease 

**Figure 2:**
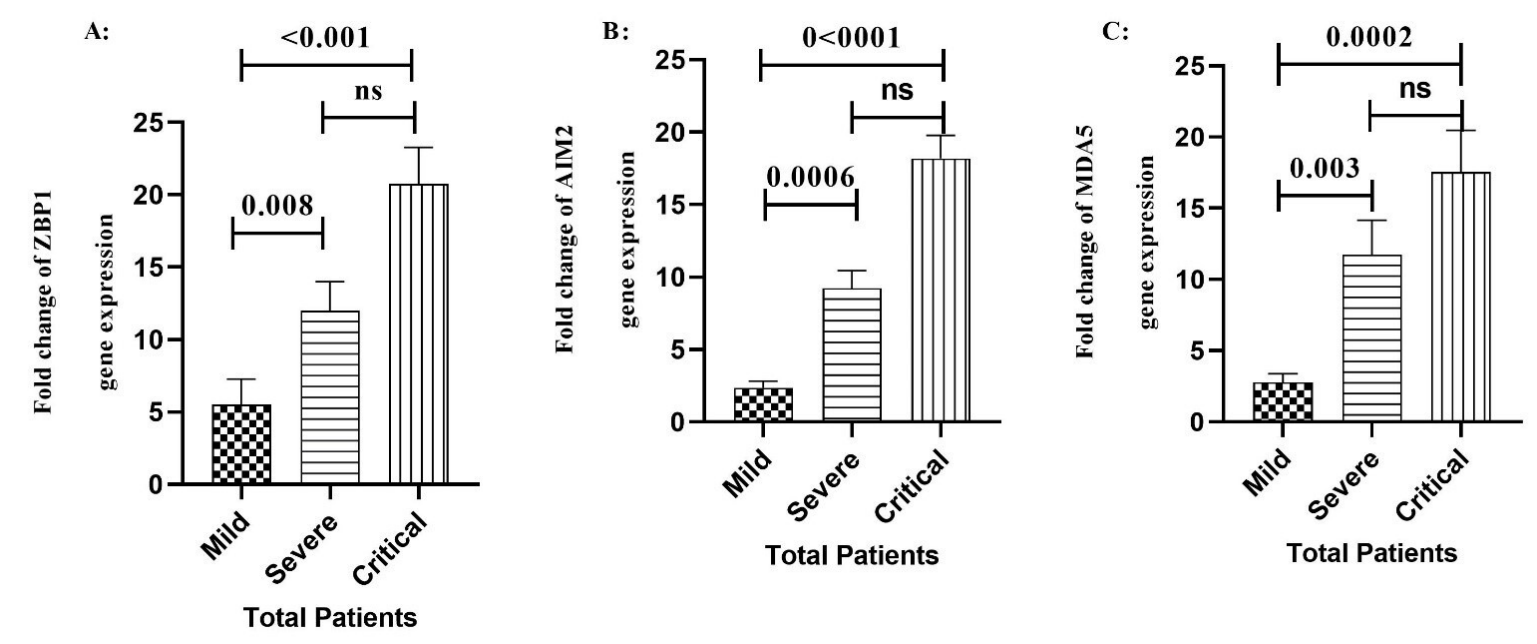
Comparison of the expression of ZBP1 A), AIM2 B), and MDA5 C) genes in different stages of the COVID-19 patients. Data are presented as fold change. ns: not significant. There are difference between specified groups (p < 0.05).

We found a significant increase in the expression level of ZBP1 gene in the critical (P<0.001) and severe (P=0.008) COVID-19 patients compared to the mild group (Figure 2A(Fig. 2)). We also found that AIM2 transcript expression level was significantly increased in the critical (P<0.001) and severe (P=0.0002) COVID-19 patients compared to the mild group (Figure 2B(Fig. 2)). Moreover, data analysis showed that the expression level of MDA5 gene was significantly higher in the critical (P=0.0006,) and severe (P= 0.003) COVID-19 patients compared to the mild group (Figure 2C(Fig. 2)).

### Comparison of the expression levels of ZBP1, AIM2, and MDA5 genes between the males and females in different stages of COVID-19 patients 

**Figure 3:**
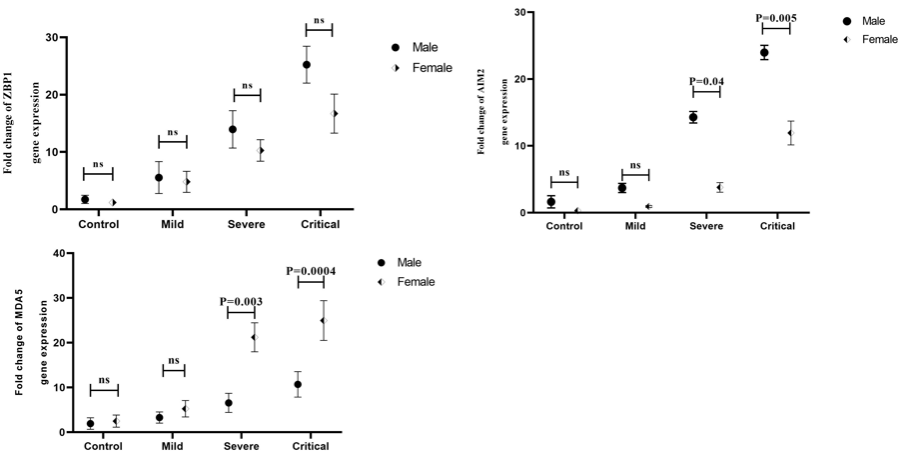
Comparison of ZBP1 A), AIM2 B), and MDA5 C) gene expression levels between male and female patients in different outcomes of the disease. Data are presented as fold change. ns: not significant. There are differences between specified groups (p < 0.05).

Remarkably, the mRNA levels of AIM2 were significantly elevated in the male critical (P=0.005) and severe (P=0.04) COVID-19 patients compared to female COVID-19 patients in the same stage (Figure 3B(Fig. 3)). Our results also showed a significant increase in the expression level of MDA5 gene in the male critical (P=0.0004) and severe (P=0.003) COVID-19 patients in comparison of the female critical and severe COVID-19 patients (Figure 3C(Fig. 3)). As the disease progressed, male ZBP1 mRNA expression was more than that of females; however, there was no significant difference in any stage of the disease (Figure 3A(Fig. 3)).

### The expression levels of ZBP1, AIM2, and MDA5 genes in the male and female COVID-19 patient groups as well as healthy control group

**Figure 4:**
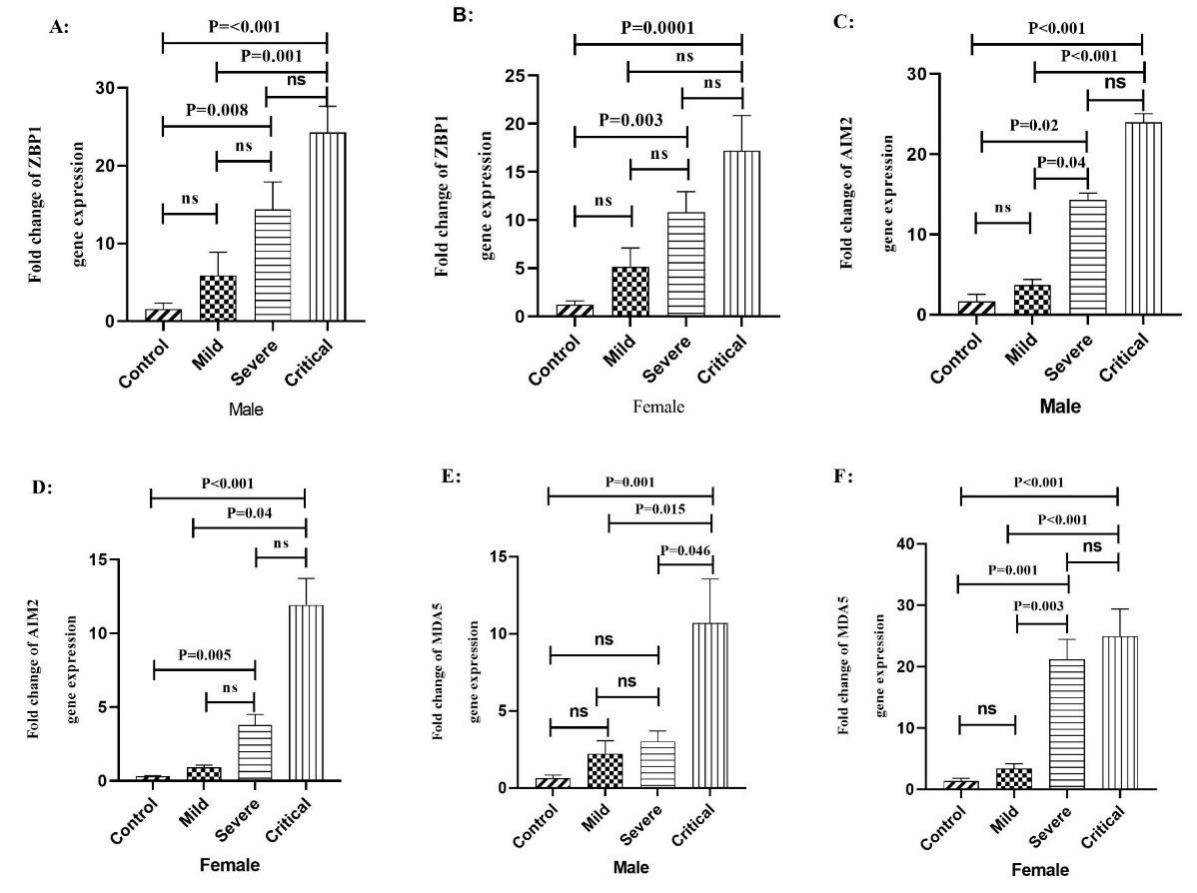
The expression level of ZBP1, AIM2, and MDA5 genes in male and female patients with COVID-19 according to the outcome of disease and gender. A) The expression level of ZBP1 gene in male COVID-19 patients. B) The expression level of ZBP1 gene in female COVID-19 patients. C) The expression level of AIM2 gene in male COVID-19 patients. D) The expression level of AIM2 gene in female COVID-19 patients. E) The expression level of MDA5 gene in male COVID-19 patients. F) The expression level of MDA5 gene in female COVID-19 patients. Data are presented as fold change. ns: not significant. There is a difference between the specified groups (p < 0.05).

The expression level of ZBP1 mRNA was significantly increased in the male critical patients compared to the mild ones (P=0.001) and healthy control subjects (P<0.001). Moreover, in severe COVID-19 patients, ZBP1 mRNA was significantly increased compared to the healthy control group (P=0.008) (Figure 4A(Fig. 4)). In addition, there were significant elevations in the levels of ZBP1 expression in the female critical (P=0.0001) and severe (P=0.003) COVID-19 patients compared to the healthy control group (Figure 4B(Fig. 4)).

We also found a significant increase in the expression level of AIM2 transcript in the male critical and severe COVID-19 patients compared to the mild (P<0.001 and P=0.04 respectively) and healthy control group (P<0.001 and P=0.02, respectively) (Figure 4C(Fig. 4)). There was also a significant increase in AIM2 mRNA in female patients with critical and mild levels (P=0.04). Additionally, we found a significant increase in the level of AIM2 transcript in the female critical (P<0.001) and severe (P=0.005) COVID-19 patients compared to the healthy control group (Figure 4D(Fig. 4)). 

In addition, MDA5 transcript in male critical patients showed a significant increase compared to the severe (P=0.04), mild (P=0.01) and control groups (P= 0.001) (Figure 4E(Fig. 4)). In addition, a significant increase was seen in the level of MDA5 transcripts in the female critical and severe COVID-19 patients compared to the mild (P=0.001 and P=0.003, respectively) and healthy control group (P<0.001 and 0.001, respectively) (Figure 4F(Fig. 4)).

## Discussion

SARS-CoV-2 is associated with COVID-19 global pandemic and has raised international concerns. It causes not only severe pneumonia and acute respiratory distress syndrome (ARDS) in vulnerable people, but also damage to many organs including the heart, kidney, pancreas, etc., and in severe cases, the disease can lead to death (Anka et al., 2021[1]; Mokhtari et al., 2020[28]). An antiviral defense initiates with the recognition of viruses by a wide range of PRRs (Kasuga et al., 2021[19]). SARS-CoV-2 could induce transcription of the genes encoding type I and III interferon (IFNs), as well as pro-inflammatory cytokines (Sampaio et al., 2021[36]). Despite the ability of IFNs to help clear the virus infection, aberrant production and impaired inflammasome activation, due to overexpression and overactivation, play a significant role in the pathogenesis of COVID-19 (Sharif-Zak et al., 2022[37]). Here, we determined and compared the expression levels of ZBP1, AIM2, and MDA5 genes transcript in different stages of COVID-19 disease as well as healthy control subjects. 

The results revealed that the expression level of ZBP1 gene in COVID-19 patients significantly increased compared to the control group. Our results also revealed an ascending increase in the expression of ZBP1 mRNA from mild to critical stage in both male and female groups (Figure 2(Fig. 2)). ZBP1 is one of the important DNA sensors that can recognize DNA and RNA molecule of viruses when they are replicating or transcribing (Maelfait et al., 2017[25]), and it is bone fide inflammation mediator by its adaptor protein (Peng et al., 2021[32]). A study reported that ZBP1 signaling pathway was involved in necroptosis by TNF (Zhu et al., 2018[53]). Li et al. demonstrated that this signaling complex could be involved in the lung damage and cause exacerbation in the conditions of COVID-19 patients (Li et al., 2020[22]). Moreover, Peng et al. reported that ZPB1, as a primary response, stimulated the inflammatory signaling pathway that depends on ubiquitination and RIPK3's scaffolding ability. They also demonstrated that ZBP1 affected the production of inflammatory cytokines, such as IP-10 and CXCL10 in critical patients as a part of the cytokine storm (Peng et al., 2021[32]). Furthermore, it has been reported that ZBP1 is a vital gene expressed following infection of human and mice lung cell with an isolate of *Middle East Respiratory Syndrome (MERS)-*CoV and SARS-CoV-1 (Mamoor, 2020[26]). Kuriakose and colleagues also showed that ZBP1-mediated sensing PB1 and NP proteins of IAV triggered the inflammatory responses and cell death through RIPK1-RIPK3-caspase-8 (Kuriakose et al., 2016[21]). During infection with IAV in mice lacking ZBP1, inflammation and epithelial damages were reduced, which led to decreased mortality (Wang et al., 2008[44]). These findings indicate a positive association between the expression level of ZBP1 mRNA and disease severity and confirm our results, showing that high levels of ZBP expression could lead to the production of inflammatory cytokines, reduction of the immune system, and progression of the disease.

Our results also showed that the level of AIM2 transcript was significantly different between the COVID-19 patients and control group (Figure 4(Fig. 4)). In this regard, the results of Junqueira's study suggested that increasing the expression level of the AIM2 gene in COVID-19 patients, as one of the important molecules, might contribute to exacerbation of the condition of hospitalized patients (Junqueira et al., 2021[15]). Other studies indicated that AIM2 by processing Gradermin D protein, as one of the essential components of the inflammasome complex and apoptosis-associated Speck-like protein containing a caspase recruitment domain (ASC) (Pontelli et al., 2020[33]), activated pro-caspase 1, Interleukin-1β (IL1β) and IL18, and induced inflammation and programmed cell death (Cridland et al., 2012[6]). In another study, researchers showed that AIM2 might play a vital role in the immunopathogenesis of COVID-19 patients including primary pneumonia to respiratory failure and systemic disease. Moreover, they found a strong association between Gradermin D and inflammation caused by inflammasome in the severe COVID-19 patients compared to those with milder disease (Junqueira et al., 2021[15]). Accordingly, these results indicated that AIM2 signaling might contribute to an unbalanced immune response in COVID-19 patients characterized by enhanced inflammatory responses, reduced antiviral signaling, potentially leading to tissue damage or disease severity.

Here, we showed that the expression of the MDA5 gene in COVID-19 patients increased compared to the control group (Figure 1C(Fig. 1)). MDA5 expression plays an substantial role in inducing antiviral responses (Junior et al., 2019[13]). Studies revealed that interferons induced by PRR play an important role in inhibiting SARS-CoV-2 infection (Paludan and Mogensen, 2022[30]). Interestingly, MDA5 also plays a critical role in the two other severe coronavirus-infected diseases that happened in this century including SARS-1 and MERS (Xu, 2020[46]). In a study conducted by Sampaio and colleagues, it was found that the IFN type I and III response to SARS-CoV-2 depended on the MDA5, a sensor that is part of the detection process of SARS-CoV2 RNA (Sampaio et al., 2021[36]). Another research group demonstrated that MDA5 was the main sensor of SARS-CoV-2 in the lung cancer cell line, Calu-3 cells (Rebendenne et al., 2021[34]). It was noted that Wang et al. reported persistent overactivation of the MDA5 signaling in severe COVID-19 patients (Bader et al., 2022[3]), which confirms our findings. A study by Wang et al. concluded that the MDA5 signaling might be persistently over-activated in severe COVID-19 patients (Wang et al., 2021[41]), which confirming our data. Accordingly, this suggests that MDA5 expression might play a protective role in COVID-19 patients by causing interferon induction in the case of SARS-CoV-2 infection.

The increased expression of ZBP1, AIM2 and MDA5 genes can be interpreted in two ways. In the first case, the expression of these genes may increase as a result of corona virus infection, and in the second case, the person may become more susceptible to COVID-19 infection as a result of the increased expression of these genes. 

The expression of ZBP1, AIM2, and MDA5 genes may increase as a result of corona virus infection. Influenza A virus (IAV)-induced expression of ZBP1 has been shown to be mediated by type-I IFN in BMDMs in vitro (Kuriakose et al., 2016[21]). Another group of researchers discovered that ZBP1 was significantly induced by pro-inflammatory mediators in lung tissues infected by IAV (Wang et al., 2019[43]). It has also been reported that IAV-induced type I IFN signaling is required for the ZBP1 upregulation (Kesavardhana et al., 2017[20]; Zheng and Kanneganti, 2020[51]). SARS-CoV-2 also infects blood monocytes, causing AIM2 inflammasomes to be activated (Junqueira et al., 2021[15]). During severe COVID-19, mitochondrial (mt) DNA and/or cell free (cf) DNA play an important role in triggering inflammatory responses and following cytokine storms, which can increase ISG mRNA such as AIM2. One study showed that oxidized mtDNA activates AIM2, which is unexpectedly activated by SARS-CoV-2 (Ferreira et al., 2021[8]). This dysregulation of mitochondrial function results in the formation of reactive oxygen species (ROS) and release of mtDNA into the cytosol of SARS-CoV-2- infected cells. ROS formation causes oxidization of mtDNA, and this oxidized mtDNA is a potent activator for AIM2 (Moriyama et al., 2020[29]). This increase in cfDNA in COVID-19 patients has been linked to the activation of the AIM2 inflammasome (Kaivola et al., 2021[16]). Furthermore, in the case of MDA5, a study reported that coronaviruses induce expression of MDA5, as previously demonstrated for mouse hepatitis virus (MHV) (Roth-Cross et al., 2008[35]). In addition, higher expression of MDA5 is triggered by SARS-CoV-2 infection, as reported by Loske et al. (2022[24]).

On the other hand, genetic variation in the regulatory and coding regions of host genes, including those involved in immune system response might play an important role in the pathogenesis and outcome of viral infections. In this regard, a gene cluster on chromosome 3 containing CCR1, CCR3, CXCR6, and CCR9, has been identified as a major risk factor for COVID-19 at the genome-wide level (Zeberg and Pääbo, 2020[48]). Furthermore, whole-exome sequencing revealed that loss-of-function variants of the X-chromosomal TLR7 in COVID-19 male patients could explain the predominance of COVID-19 (van der Made et al., 2020[40]). Variants at the TLR3 and IRF7 genes have also been linked to life-threatening COVID‐19 pneumonia and asymptomatic infection. Moreover, Zhang et al. found that at least 3.5 % of patients with life-threatening COVID-19 pneumonia had known or new genetic defects at eight of the candidate loci involved in the TLR3- and IRF7-dependent induction and amplification of type I IFNs (Zhang et al., 2020[49]). 

Regarding the subjects' gender, data analysis showed that the expression level of AIM2 and MDA5 mRNA was significantly increased in the male critical and severe COVID-19 patients than females in the same stage. Moreover, the expression level of ZBP1, AIM2, and MDA5 genes was different between the male critical patients and male mild and healthy subjects (Figure 4A(Fig. 4)). In addition, the expression level of AIM2 and MDA5 genes was higher in the female severe and critical groups than healthy control groups (Figure 4D, F(Fig. 4)). A noticeable feature of the SARS-CoV-2 infection was the difference in the severity and outcome of COVID-19 disease between males and females (Galasso et al., 2020[9]; Jin et al., 2020[12]; Peckham et al., 2020[31]). Also, male patients tended to display more inflammation responses that contributes to the disease progression and outcome (Jin et al., 2020[12]; Peckham et al., 2020[31]). Furthermore, studies revealed that the odds of hospitalization, ICU admission, and mortality were nearly three times higher in male patients compared to females (Chan et al., 2020[4]). Although the underlying mechanisms are unclear, several factors have been speculated to account for the disparity including differences in biology, behavior, occupation, etc. (Galasso et al., 2020[9]). In addition, previous studies have shown that immune responses in females have been more effective than males against pathogens such as SARS-CoV-2 (Pontelli et al., 2020[33]; Singer, 2019[38]). This might be due to the effects of sex hormones, genes escaping X chromosome inactivation, skewed X chromosome inactivation, miRNAs encoded on the X chromosome, and the varied expression of DNA Methyl Transferase (DNMTs) in males and females (Migliore et al., 2021[27]). Another possible reason for the decreased transcription of MDA5 in the male subjects might be the role of SARS-CoV-2 proteins which could regulate antiviral responses. In this regard, researchers explain that the SARS-CoV-2 membrane protein (M) interacts with MDA5 and inhibits the production of type I and III IFNs by preventing the multiprotein complex containing MAVS, TRAF3, and TBK1 (Zheng et al., 2020[51]). In conclusion, the results of our study showed a steady increase in the expression of ZBP1, AIM2, and MDA5 genes transcript, indicating that ZBP1, AIM2, and MDA5 might play important roles in the severity and outcome of COVID-19 disease. Moreover, the expression level of ZBP1, AIM2, and MDA5 was higher in male patients than females, suggesting that the severity of COVID-19 disease in male patients compared with female ones might be related to the higher expression of AIM2 and MDA5. We recommend that further studies be conducted to clarify this issue.

## Notes

Ramin Yaghobi and Jamal Sarvari (Department of Bacteriology and Virology, School of Medicine, Shiraz University of Medical Sciences, Shiraz, Iran; Phone: +9871-42652781, FAX: +9871-42710780, Email: sarvarij@sums.ac.ir) contributed equally as corresponding author.

## Declaration

### Conflict of interest

All authors declare no conflict of interest.

### Authors' contribution

Study concept: Sarvari J, Yaghobi R, Arab Zadeh SAM and Farokhnia M; Study design: Sarvari J, Yaghobi R and Ramezani A; Bench work: Arefinia N; Patients selection: Farokhnia M; Data analysis: Arefinia N and Ramezani A; Manuscript drafting: Arefinia N; Critical revision of the manuscript: Sarvari J, Yaghobi R, Arab Zadeh SAM, Farokhnia M and Ramezani A. All authors read and approved the final manuscript.

### Ethical approval 

Written informed consent was obtained from each participant, and the study was approved by the local Ethics Committee of Shiraz University of Medical Sciences (IR.SUMS.REC.1400.737).

## Figures and Tables

**Table 1:**
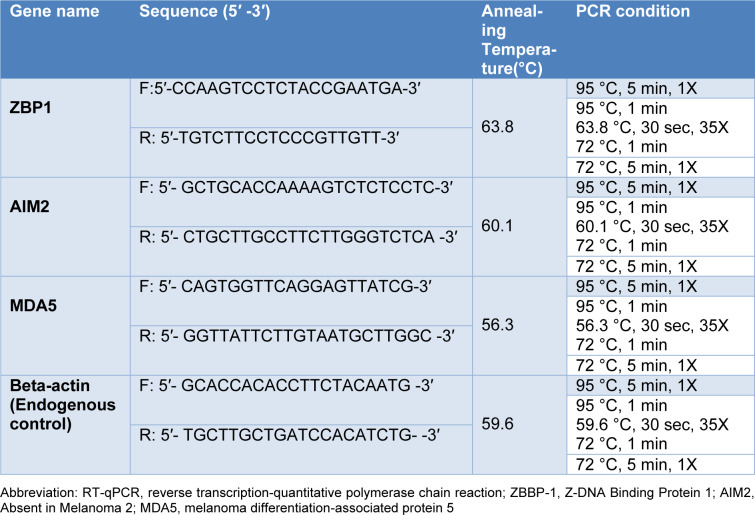
The sequences of primers used for RT-qPCR in the study

**Table 2:**
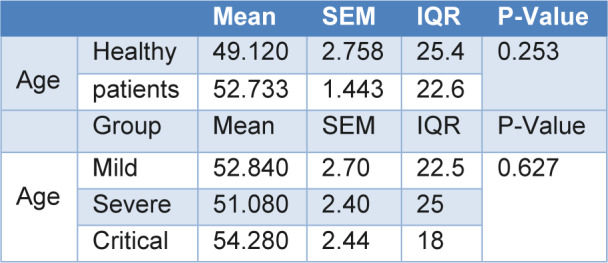
Demographical characteristics of the three study groups and healthy volunteer control group
